# Multifunctional cytokine production marks influenza A virus‐specific CD4 T cells with high expression of survival molecules

**DOI:** 10.1002/eji.202350559

**Published:** 2023-07-30

**Authors:** Lotus M. Westerhof, Jonathan Noonan, Kerrie E. Hargrave, Elizabeth T. Chimbayo, Zhiling Cheng, Thomas Purnell, Mark R. Jackson, Nicholas Borcherding, Megan K. L. MacLeod

**Affiliations:** ^1^ School of Infection and Immunity University of Glasgow Glasgow UK; ^2^ Baker Heart and Diabetes Institute & Baker Department of Cardiometabolic Health University of Melbourne Melbourne Australia; ^3^ Malawi Liverpool Wellcome Centre Blantyre Malawi; ^4^ School of Cancer Sciences University of Glasgow Glasgow UK; ^5^ Department of Pathology and Immunology Washington University St Louis Missouri USA

**Keywords:** CD4 T cells, Cytokine, Immune memory, Influenza virus, Survival

## Abstract

Cytokine production by memory T cells is a key mechanism of T cell mediated protection. However, we have limited understanding of the persistence of cytokine producing T cells during memory cell maintenance and secondary responses. We interrogated antigen‐specific CD4 T cells using a mouse influenza A virus infection model. Although CD4 T cells detected using MHCII tetramers declined in lymphoid and non‐lymphoid organs, we found similar numbers of cytokine^+^ CD4 T cells at days 9 and 30 in the lymphoid organs. CD4 T cells with the capacity to produce cytokines expressed higher levels of pro‐survival molecules, CD127 and Bcl2, than non‐cytokine^+^ cells. Transcriptomic analysis revealed a heterogeneous population of memory CD4 T cells with three clusters of cytokine^+^ cells. These clusters match flow cytometry data and reveal an enhanced survival signature in cells capable of producing multiple cytokines. Following re‐infection, multifunctional T cells expressed low levels of the proliferation marker, Ki67, whereas cells that only produce the anti‐viral cytokine, interferon‐γ, were more likely to be Ki67^+^. Despite this, multifunctional memory T cells formed a substantial fraction of the secondary memory pool. Together these data indicate that survival rather than proliferation may dictate which populations persist within the memory pool.

## Graphical abstract

Protective memory CD4 T cells must be functional and long lived. Memory CD4 T cells producing multiple cytokines expressed high levels of pro‐survival molecules. These cells proliferated poorly following re‐infection but are stably maintained. This suggests that different cytokine^+^ memory CD4 T cell populations have distinct roles to protect the host.

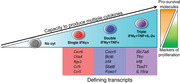

## Introduction

Immunological memory protects against repeated infection with a strong and rapid pathogen‐specific response with cytokine production often central to protection [1, 2, 3, 4, 5, 6, 7, 8, 9]. Improving our understanding of T cell memory is particularly important in the context of highly variable infections such as influenza A virus (IAV). Although neutralising antibodies can prevent IAV infection, frequent mutations within viral surface proteins make it difficult for strain‐specific antibodies to recognise altered viruses [10].

In contrast, CD4 and CD8 T cells recognise conserved IAV epitopes [11, 12]. The presence of cytokine producing IAV‐specific CD4 and CD8 T cells in human peripheral blood correlates with cross‐strain protection against symptomatic influenza disease [13, 14, 15, 16]. Mouse studies have demonstrated protection by CD4 and CD8 T cells, with the inflammatory T‐helper (Th1) cytokine, interferon (IFN)‐γ, often essential [2, 4, 6, 8]. The cytokines TNF and interleukin (IL‐) 2 are also implicated in protection to IAV [17, 18]. These data support that the most effective memory T cells are those with the capacity to produce cytokines.

Multifunctional T cells, capable of producing a number of different cytokines, have been associated with effective immune protection in human disease and animal models [1, 3, 5, 9]. It remains unclear, however, why these cells provide enhanced protection compared to other populations. These data suggest that vaccines that drive cytokine producing T cells would be the most protective. However, although some studies found that cytokine producing T cells can become memory cells, others have described limited persistence of these cells [19, 20, 21, 22].

We previously showed that multifunctional CD4 and CD8 T cells expressing IFN‐γ, IL‐2 and TNF increase in predominance from the primary to the memory pool [23]. These data supported the conclusion that a rapid production of cytokines does not limit long‐term immunity. Building on this, we have now tracked the characteristics of the IAV‐specific T cell pool at multiple time points post‐infection. Our data show that CD4 T cells with the capacity to produce cytokine express higher levels of pro‐survival molecules than cells that do not produce cytokines. Multifunctional CD4 T cells, in particular, express a number of genes associated with cell survival. Following a re‐challenge infection, multifunctional T cells expressed low levels of Ki67, a marker of proliferation, compared to other cytokine^+^ T cell populations. Despite this, the proportion of cytokine^+^ cells that were multifunctional did not alter following the re‐infection. Together these data demonstrate that the capacity to produce cytokine does not limit persistence of CD4 T cells into the primary or secondary memory pool.

## Results

### MHC tetramers and cytokine expression enable analysis of IAV‐specific T cells

**Figure 1:**
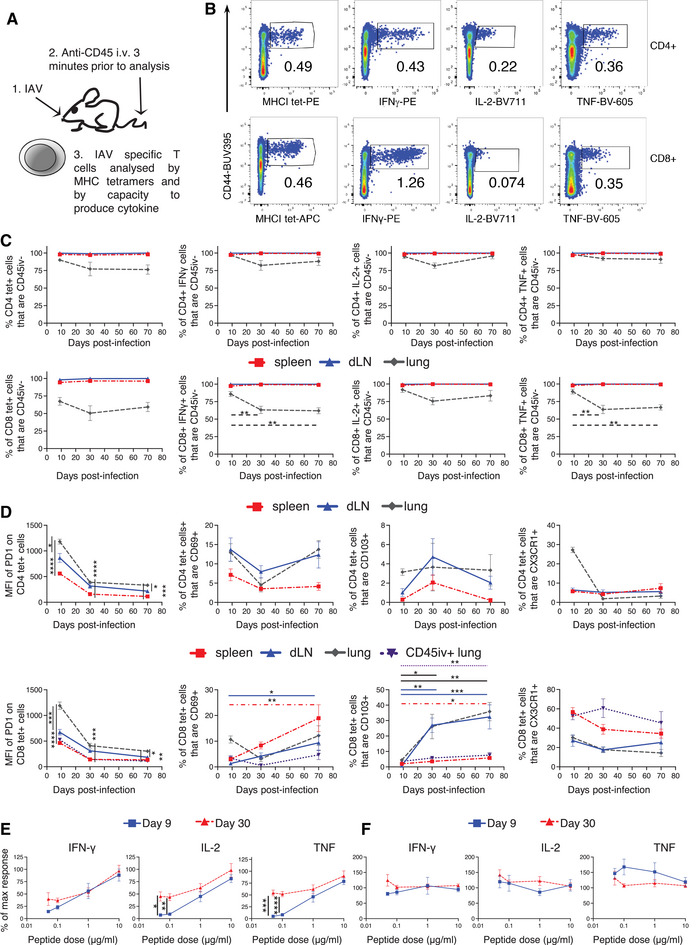
*MHC tetramers and cytokine expression enable the analysis of influenza A virus (IAV)‐specific T cells*. C57BL/6 mice were infected i.n. with IAV on day 0 and injected i.v. with fluorescently labelled anti‐CD45 3 min prior to removal of organs (A). Single‐cell suspensions of spleens, mediastinal draining LN (dLN) and lung were examined 9, 30 or 70 days post‐IAV infection. Cells were either stained with IA^b^/NP_311–325_ or D^b^/NP_368–374_ tetramers, or cells were restimulated for 6 h with dendritic cells (DCs) presenting NP_311–325_ and NP_368–374_ peptides. Example flow cytometry plots of splenic IAV‐specific cells as gated in Supporting Information Fig. 1A (B). Percentages of IAV‐specific cells that bound to the i.v. injected anti‐CD45, cells gated as in Supporting Information Fig. 1B (C). The phenotype of MHC tetramer^+^ CD4 and CD8 T cells (D). Spleen cells from IAV‐infected mice restimulated with DCs cultured with 0.05, 0.1, 1, 10 or 100 mg/mL of NP_311–325_ and NP_368–374_ (E and F). The percentage of max cytokine production as detected at 100 mg/mL was calculated. In (B–D), data are from two time course experiments with a total of 7–8 mice/time point. In (C and D), symbols show the mean of the group, and error bars are SEM. Statistical difference tested by ANOVA followed by a Dunnett's multiple comparison test for analyses between time points and by a Tukey's test between organs. In (E and F), data are from two experiments with a total of 6–8 mice per time point. Significance between primary and memory tested by ANOVA followed by a Šídák's multiple comparison test. *: *p* < 0.05, **: *p* < 0.01, ***: *p* < 0.001, ****: *p* < 0.0001.

We used MHCI and MHCII tetramers (tet) containing immunodominant IAV nucleoprotein (NP) peptides (NP_368–374_ and NP_311–325_) to identify and characterise responding T cells longitudinally following infection (Fig. 1A and B; Supporting Information Fig. 1). These peptides were also used to activate cells from the same mice to examine the anti‐IAV T cell cytokine responses (Fig. 1B), as optimal dual staining of MHC tetramers and cytokines was not possible.

IAV infected animals were injected with fluorescently labelled anti‐CD45 shortly before euthanasia to label cells in the blood. The majority of lymphoid organ MHC tet^+^ and cytokine^+^ CD4 and CD8 T cells and lung CD4 T cells were negative for CD45iv, whereas around half of the lung IAV‐specific CD8 T cells were within the blood (Fig. 1C), cells gated as shown in Supporting Information Fig. 1B.

Although PD1 decreased on all the IAV‐specific T cells after day 9, T cells in the lung expressed the highest levels at all time points (Fig. 1D). Tissue resident memory associated markers, CD69 and CD103 [24], increased on CD8 MHCI tet^+^ cells in the lymphoid organs and lung CD45iv negative lung cells after day 9. However, very few CD4 MHCII tet^+^ cells expressed these markers.

Although at day 9, some CD4 MHCII tet^+^ cells in the lung expressed CX3CR1, only a small minority of memory cells expressed this chemokine receptor. CX3CR1 was expressed by CD8 MHCI tet^+^ cells, most prominently in the spleen and on lung CD45iv^+^ cells suggesting that these are circulating effector memory cells [25].

In addition to phenotypic changes, cytokine^+^ memory CD4 T cells displayed increased sensitivity to peptide compared to primary responding cells (Fig. 1E). In contrast, primary and memory CD8 T‐cell cytokine production was unaffected by peptide dose (Fig. 1F).

### While the number of IAV‐specific CD4 T cells detected by MHC tetramer decline from day 9 to 30, the numbers of cytokine^+^ CD4 T cells in the secondary lymphoid organs are stable

We examined the numbers of T cells detected by MHC tetramers or IFN‐γ production between days 9 and 30 post‐infection. As MHC tetramers detect NP‐specific T cells regardless of their ability to produce cytokines, altered dynamics between these and cytokine^+^ cells suggest differences between cells that can and cannot produce cytokine.

**Figure 2:**
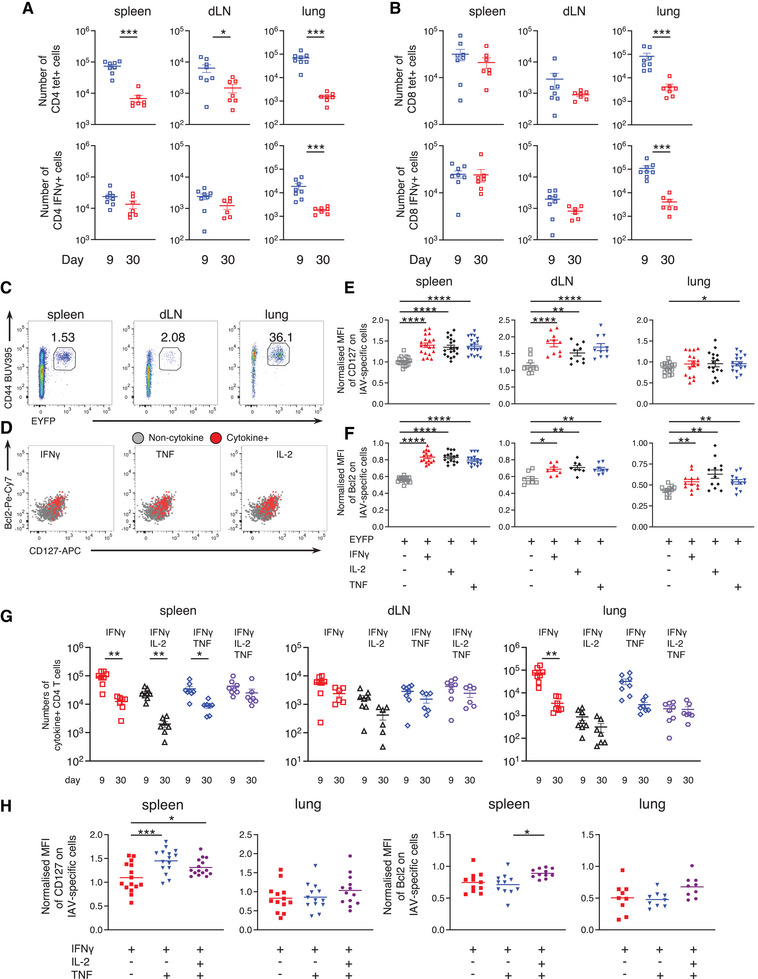
*Influenza virus‐specific cytokine* ^+^ *CD4 T cells are more likely to enter the memory pool than non‐cytokine* ^+^ *cells*. C57BL/6 (A and B, G) or TRACE (C–F, H) mice were infected i.n. with influenza A virus (IAV) on day 0 and injected i.v. with fluorescently labelled anti‐CD45 (CD45iv) 3 min prior to removal of organs. Single‐cell suspensions of spleens, mediastinal draining LN (dLN) and lung were examined 9 or 30 days post‐IAV infection and either stained with MHCII/nucleoprotein (NP) or MHCI/NP tetramers or activated with IAV‐peptide loaded dendritic cells (DCs) (A and B) or IAV‐Ag loaded DCs (C–H). Numbers of MHC tetramer or IFN‐γ^+^ CD4 (A) or CD8 T cells (B). Example FACS plots from the indicated organs of TRACE mice (C) and the spleen (D) are gated on live CD4^+^ dump negative, CD45iv negative cells (C) and on EYFP^+^ cytokine negative: grey; IFN‐γ, IL‐2 and/or TNF^+^: red (D). In (A and B) and (E–H), symbols represent a mouse, and the lines shows the means, error bars are SEM. In (A and B) and (G), data are from two independent time course experiments with a total of 7–8 mice/time point, significance tested by a Mann–Whitney. In (E and F) and (H), data are from three independent experiments with a total of 5–8 mice and normalised by dividing the MFI on EYFP^+^ or cytokine^+^ cells by the MFI of naïve CD44^lo^ CD4 T cells from cells from the same mouse and organ. In (E–H), samples from some dLNs and lungs were excluded as the numbers of cytokine^+^ cells collected were too low for analysis. Significance tested via a Friedman paired analysis with Dunn's multiple comparison test, *: *p* < 0.05, **: *p* < 0.01, ****: *p* < 0.0001.

The numbers of IAV‐specific CD4 T cells detected by MHCII tet were reduced between days 9 and 30 in all three organs (Fig. 2A). In contrast, the numbers of CD4 T cell detected by cytokine production did not decline in the spleen and draining LN (dLN) (Fig. 2A, Supporting Information Fig. 2A). Although the numbers of IFN‐γ^+^ and TNF^+^ cells were lower in the lung at day 30 than day 9, the numbers of IL‐2^+^ cells were stable. In contrast, the numbers of IAV‐specific CD8 T cells detected by either MHCI tetramers, IFN‐γ or TNF, remained stable in secondary lymphoid organs, although in all cases, these cells declined in the lung (Fig. 2B, Supporting Information Fig. 2B). Although the numbers of IL‐2^+^ CD8 T cells were low, the cell numbers were unchanged in the spleen and lung but did drop in the dLN between days 9 and 30.

These data suggest that CD4 T cells with the capacity to produce cytokine are more likely to enter the memory pool than those that cannot produce cytokine. To investigate this, we compared the phenotype and function of different memory CD4 T cell populations taking advantage of a reporter mouse we developed [26]. This reporter tool, TRACE, includes three transgenes to drive permanent expression of EYFP in activated T cells. The first transgene is the upstream 8.389 kb of the *Il2* promoter which drives expression of rtTA. This promoter section was selected based on findings by Yui *et al*. who characterised the *Il2* promoter, revealing DNase‐1 hypersensitivity sites in this region following TCR stimulation [27]. Only in the presence of doxycycline, can rtTA drive expression at the tet‐ON promoter. This leads to the production of Cre recombinase and subsequent removal of a stop codon at the ROSA locus. Removal of this stop codon enables permanent EYFP expression (Supporting Information Fig. 3).

As the EYFP^+^ CD4 T cells likely respond to an array of IAV antigens (IAV Ag), we used BM derived dendritic cells (DCs) cultured with a sonicated IAV Ag preparation to restimulate the T cells *ex vivo* for cytokine analysis [23]. EYFP^+^ CD4 T cells only produced cytokine following culture with DCs incubated with IAV Ag, demonstrating that cytokine production is antigen‐specific (Supporting Information Fig. 3D). By gating on CD4 T cells that produced either IFN‐γ, IL‐2 or TNF following the culture, we determined that between 49 and 69% of the cytokine^+^ cells were EYFP^+^ (Supporting Information Fig. 4A and B). This suggests that TRACE mice enable us to examine over half of the cytokine^+^ T cells that respond to IAV. Between 2 and 15% of the EYFP^+^ cells produced cytokine, a similar range as described to a model antigen inserted into IAV [28] (Supporting Information Fig. 4C and D). Importantly, the proportion of IFN‐γ^+^ CD4 T cells that also produce IL‐2 and/or TNF are similar when gating on total IFN‐γ^+^ or EYFP^+^ IFN‐γ^+^ cells (Supporting Information Fig. 4E).

The expression of the pro‐survival molecules CD127 and Bcl2 [29] was examined in EYFP^+^ cytokine negative CD4 T cells and those expressing IFN‐γ, IL‐2 or TNF 40 days post‐infection (Fig. 2C–F). In the secondary lymphoid organs, the cytokine^+^ EYFP^+^ T cells expressed higher levels of both molecules compared to cytokine negative cells. These differences were less clear in the lung, potentially reflecting the reduced survival of cytokine^+^ and negative cells in this organ. These data are consistent with the hypothesis that CD4 T cells with the capacity to produce cytokines have an enhanced survival capacity compared to non‐cytokine^+^ T cells.

Additionally, we analysed CD4 IFN‐γ^+^ cells that were also positive for IL‐2, TNF, all three cytokines, or only expressed IFN‐γ. The numbers of triple cytokine^+^ CD4 T cells did not change in any of the organs between days 9 and 30 (Fig. 2G). The numbers of all other populations declined significantly in at least one of the organs. Although triple cytokine^+^ cells in the spleen expressed high levels of CD127 and Bcl2, no differences in expression of these survival molecules were found in lung cytokine^+^ CD4 T cells (Fig. 2H). These data suggest that survival, at least based on these two molecules alone, may not explain the differences in stability of the cytokine^+^ cell populations between days 9 and 30.

### Single IFN‐γ^+^ CD4 and CD8 T cells are more likely to be in cell cycle than multifunctional T cells

**Figure 3:**
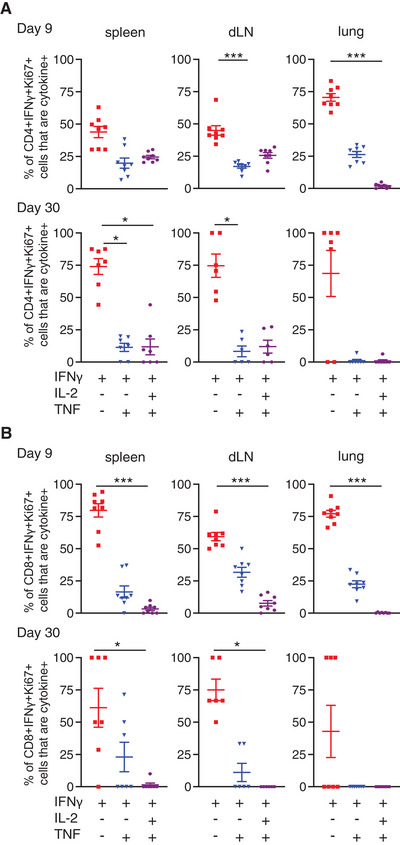
*Single IFN‐γ^+^ CD4 and CD8 T cells are more likely to be in cell cycle 9 and 30 days after influenza A virus (IAV) infection than T cells producing multiple cytokines*. C57BL/6 mice were infected i.n. with IAV on day 0 and injected i.v. with fluorescently labelled anti‐CD45 3 min prior to removal of organs at the indicated time point. Single‐cell suspensions of spleens, mediastinal draining LNs (dLN) and lungs from IAV infected mice were activated by IAV‐Ag^+^ dendritic cells (DCs) for 6 h and CD4 (A) and CD8 (B) T cells analysed. Data are from two independent time course experiments with a total of 7–8 mice/time point. Each symbol represents a mouse, and the horizontal line shows the mean of the group and significance tested via paired Friedman analysis with Dunn's multiple comparison test *: *p* < 0.05, ***: *p* < 0.001.

An alternative explanation for the stability of the numbers of triple cytokine^+^ T cells could be increased proliferation in comparison to other cytokine^+^ populations. We gated on IFN‐γ^+^ Ki67^+^ cells to examine whether these cells were producing one or more cytokine (Supporting Information Fig. 5). IFN‐γ single^+^ CD4 and CD8 T cells were more likely to be in cell cycle compared to triple^+^ cells at both days 9 and 30 (Fig. 3A and B). These data suggest that enhanced proliferation by multifunctional T cells does not explain their increase predominance in the memory pool [23] nor the lack of decline in the numbers of these cells between days 9 and 30 post‐infection (Fig. 2G).

### scRNAseq analysis reveals heterogeneity in cytokine^+^ and cytokine negative memory CD4 T cells

To obtain a more detailed understanding of differences between the cytokine^+^ and negative memory T cell populations, we performed single‐cell gene transcription analysis. TRACE mice were infected with IAV, and after 40 days, the mice were injected with anti‐CD45iv 3 min prior to tissue harvest. CD45iv negative spleen and lung CD4 T cells were isolated and activated with IAV‐Ag^+^ DCs for 4 h to drive cytokine mRNA expression. We sorted EYFP^+^ cells from the spleens and lungs and, to provide a base line level of gene expression, naïve, CD44^lo^ EYFP‐negative CD4 T cells from the spleen (Supporting Information Fig. 6). Cells from four infected TRACE mice were combined with oligo tagged antibodies used to distinguish each animal and organ.

**Figure 4:**
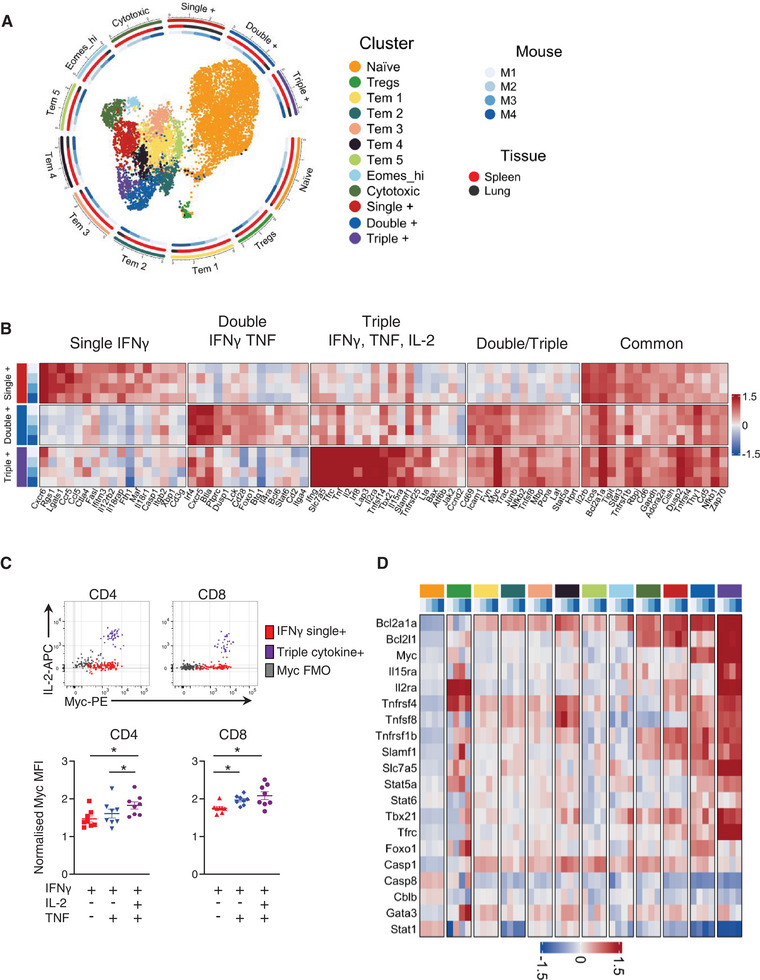
*Triple cytokine* ^+^ *CD4 T cells have a pro‐survival transcriptional signature*. TRACE mice were infected with influenza A virus (IAV) and 40 days later injected with anti‐CD45 3 min prior to removal of spleens and lungs. Isolated CD4 T cells were activated for 4 h with IAV‐Ag dendritic cells (DCs) and the CD45iv negative CD4 + CD44^hi^ EYFP^+^ cells (spleens and lung) and CD4 + CD44^lo^ EYFP‐negative (spleens) cells were FACS sorted and their transcriptomics examined by scRNAseq. Uniform Manifold Approximation and Projection (UMAP) of 1 naïve cluster, 1 Treg cluster and 10 memory clusters. The radial tracks around the UMAP reflect the numerical representation of each cluster, and the mouse/tissue origin within each cluster on a logarithmic axis (A). DEGs in single, double and triple cytokine^+^ clusters identified by comparison between the indicated populations and all other clusters (B). The normalised MFI of Myc by single, double and triple cytokine^+^ cells in CD4 and CD8 T cells from C57BL/6 IAV infected mice examined at day 40 post‐infection; expression was normalised by dividing each sample's MFI by the MFI on naïve CD4^+^ cells in the same sample (C). Expression of mRNA for cell survival molecules from the scRNAseq analysis (D, legend as in A). Data in (A, B, D) are from one experiment with cells from four mice. In (C), data are from two independent experiments with each symbol representing one mouse and the horizontal line showing the mean of the groups; significance tested by paired ANOVA with Tukey's multiple comparison test, *: *p* < 0.05.

Initial clustering produced 19 different clusters, including 8 naïve clusters, 10 memory clusters and one Treg cluster (Supporting Information Fig. 7A). To focus on the differences between the memory clusters, we reduced the complexity of the naïve population into a single population (Fig. 4A); all clusters remained transcriptionally distinct (Supporting Information Fig. 7B; Supporting Information Table 1). Of the 10 memory clusters, 7 expressed little to no *Ifng*, *Tnf or Il2* (Supporting Information Fig. 7C). Cells from all memory clusters were found in both the spleen and lung, with the spleen contributing most cells in all but the *Ifng* ^+^ cluster (Fig. 4A; Supporting Information Fig. 7D). Naïve and Treg clusters cells were almost entirely from the spleen, consistent with their origin from the naïve sort gate (Supporting Information Fig. 6).

Triple^+^ cells were uniquely enriched for *Il2* and, whereas the single^+^ and double^+^ clusters clearly expressed *Ifng* ^+/−^ *Tnf*, these transcripts were only significantly enriched (versus all other cells) within the triple^+^ cluster. Previous studies have demonstrated that multifunctional T cells produce more IFN‐γ on a per cell basis than single IFN‐γ^+^ T cells [1, 3, 5]. We also found that triple cytokine^+^ T cells express the greatest amounts of IFN‐γ by flow cytometry (Supporting Information Fig. 8). Moreover, by gating on total IL‐2^+^ or TNF^+^ T cells, we found that triple cytokine^+^ CD4 T cells also expressed more of these cytokines than single IL‐2^+^ or TNF^+^ cells, respectively.

### Triple cytokine^+^ memory CD4 T cells express a pro‐survival gene signature

We focussed on the transcriptional signatures of the three cytokine producing clusters: single^+^ (*Ifng*) double^+^ (*Ifng* and *Tnf*) and triple^+^ (*Ifng, Tnf* and *Il2*) cells (Fig. 4B). The single^+^ CD4 T cells expressed the highest levels of *Icos* and *Pdcd1* (PD1), which we confirmed at protein level (Supporting Information Fig. 9). Genes uniquely up‐regulated in the double^+^ cells included *Cxcr5*, *Bcl6, Irf4* and *Foxo1*, indicating a Tfh‐like phenotype [30, 31, 32].

We also observed genes commonly enriched across some or all cytokine^+^ clusters. Strikingly, the double and triple cytokine^+^ cells expressed high levels of the transcription factor *Myc*; we confirmed increased Myc protein expression in triple^+^ cells by flow cytometry (Fig. 4C). The triple cytokine^+^ memory CD4 T cells also expressed high levels of molecules associated with survival (Fig. 4D). These included Bcl2 family members (*Bcl2a1a* and *Bcl2l1*(Bclx)), receptors for pro‐survival cytokines (*Il2ra*, *Il15ra*) and costimulatory molecules (*Tnfrsf4* (OX40), *Tnfsf8* (CD30L)) [33, 34, 35, 36, 37, 38, 39].

### TCR clones are found in multiple memory T‐cell clusters but are not shared among different animals

**Figure 5:**
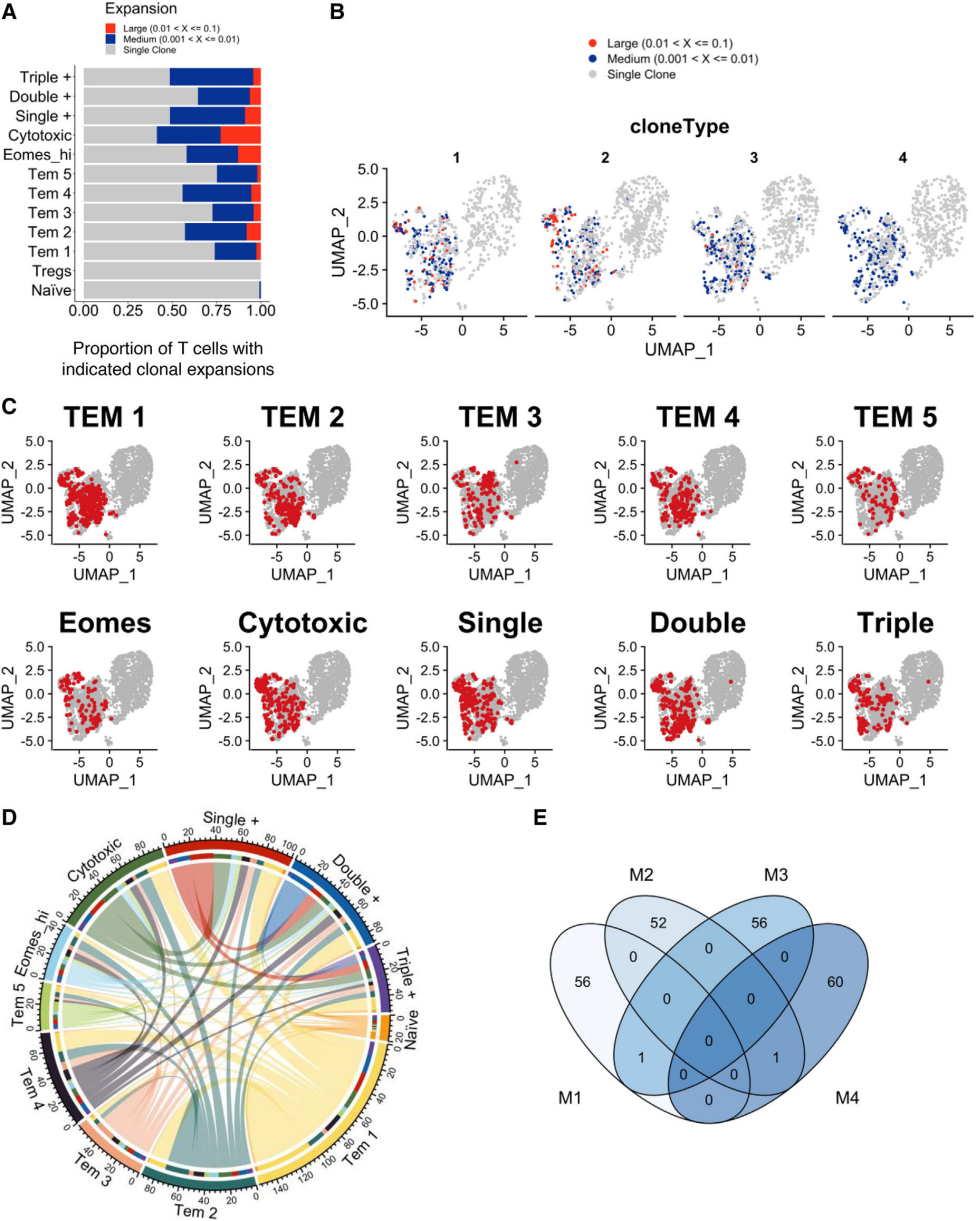
*TCR clones are found in multiple memory T‐cell clusters but are not shared between different animals*. scRNAseq analysis described in this figure was integrated with single‐cell TCR CDR3 analysis. Large, medium and single clones identified by TCR CDR3 sequencing are displayed for each cluster and the proportion of T cells with the indicated clonal expansions shown (A). Enriched clones are overlaid on the scRNAseq Uniform Manifold Approximation and Projection (UMAP) for each animal and coloured by the magnitude of expansion (B). To highlight the extent to which clones were shared across clusters, clones found within each memory cluster were identified and displayed on the UMAP (C). To provide higher resolution, these data are presented as a chord diagram, highlighting the inter‐cluster relationship of individual clones (D). The limited overlap of identified clones between mice is displayed as a Venn diagram (E).

We used CDR3 sequencing to address whether clones were restricted to particular memory T cell clusters. We found expanded clones within each of the memory populations, with the relative absence of these in the naïve and regulatory populations (Fig. 5A and B). Few expanded clones appeared cluster specific, with most represented across multiple clusters (Fig. 5C and D). These data suggest that TCR CDR3 sequence does not dictate T‐cell fate. Finally, the analysis of expanded clonotypes derived from each animal demonstrated little evidence of public T cell clones (Fig. 5E).

### Single IFN‐γ^+^ T cells dominate the proliferative response during re‐infection

Our previous data and the relationship among T‐cell multifunctionality, persistence into the memory pool, and protection from disease suggest that triple cytokine^+^ T cells should dominate a secondary response to IAV [1, 3, 5, 23, 40, 41]. To test this, we re‐infected IAV‐memory mice with a distinct strain of IAV, X31. In this model, T‐cell responses are associated with immune protection [4], and re‐challenged mice lost less weight than primary infected animals (Supporting Information Fig. 10A).

**Figure 6:**
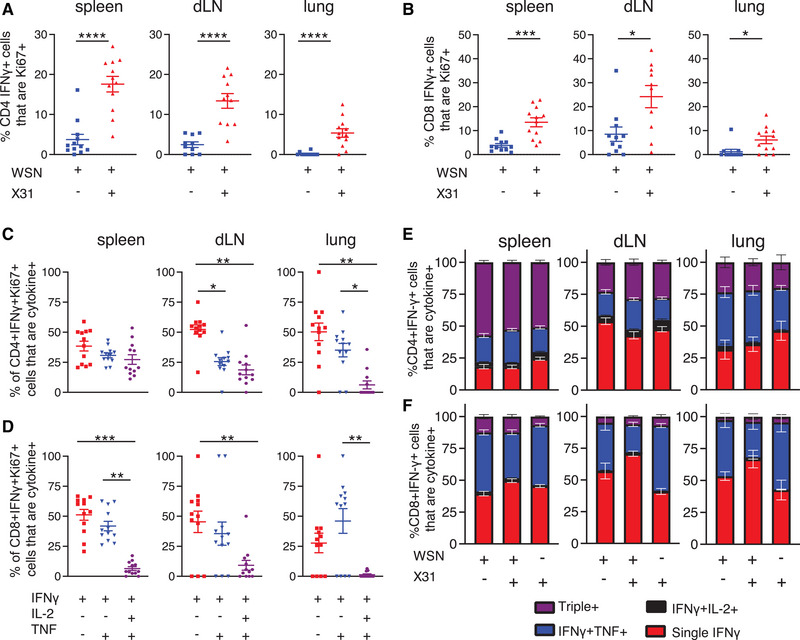
*Triple cytokine* ^+^ *T cells are less likely to be in cell cycle than single IFN*‐γ^+^ *T cells following challenge infection*. C57BL/6 mice were infected i.n. with influenza A virus (IAV) on day 0. On day 30, some of these animals and age‐matched naïve mice were infected with X31 IAV i.n. A period of 5 (A–D) or 35 days (E–F) later, these mice and the remaining WSN‐memory mice received anti‐CD45iv 3 min prior to tissue harvest. Single‐cell suspensions of spleens, mediastinal draining LN (dLN) and lung were activated for 6 h with IAV‐Ag^+^ dendritic cells (DCs). CD45iv negative IAV‐specific IFN‐γ^+^ CD4 T cells or CD8 T cells were analysed by flow to detect IFN‐γ^+^ T cells that were Ki67^+^. In (C and D), IFN‐γ^+^ that were Ki67 in the re‐infected animals at day 5 post‐infection were examined for the production of IL‐2 and TNF. Data are from two independent experiments with a total of 7–8 mice. Symbols represent each mouse, and the horizontal line shows the mean of the group (A–D), in (E and F), error bars are SEM. In (A–D), significance tested by Mann–Whitney. In (E and F), significance tested via ANOVA and Dunn's; *: *p* < 0.05, **: *p* < 0.01, ***: *p* < 0.001, ****: *p* < 0.0001.

Re‐infection led to an increase in the percentages of cytokine^+^ CD4 and CD8 T cells that expressed Ki67, most notably in cells in the spleen and dLN (Fig. 6A and B). The IFN‐γ^+^ Ki67^+^ CD4 and CD8 T cells present in the re‐infected animals were most likely to be within the single IFN‐γ^+^ population, especially in the dLN and the lung (Fig. 6C and D).

The low levels of Ki67 in triple cytokine^+^ cells may suggest that this population would be less prevalent following re‐infection and that single or double positive T cells may become a more prominent population. This was not the case. The percentages of IFN‐γ^+^ cells that were also expressing IL‐2 and/or TNF were the same before and at day 5 (Supporting Information Fig. 10B), and day 35 following re‐infection (Fig. 6E and F). These data are consistent with the hypothesis that multifunctional T cells have an ability to survive within the memory pool.

## Discussion

Our data demonstrate that CD4 T cells with the capacity to produce multiple different types of cytokines are found at stable numbers in secondary lymphoid organs across primary and memory time points. Moreover, at gene and protein level, these cells express molecules associated with cell survival. Cytokines are often required for, or associated with, memory T cell mediated immune protection [1, 2, 3, 4, 5, 6, 7, 8, 9]. Therefore, the identification of factors and cell types that promote cytokine^+^ cells could define how we could increase the size and protective ability of the memory T‐cell pool following vaccination.

Our data are consistent with the hypothesis that memory T cells with the capacity to produce cytokines are more likely to survive into and within the memory pool than those that cannot produce cytokines. This challenges findings that showed poor survival of cytokine^+^ cells and supports other studies that have demonstrated the stability of cytokine^+^ memory T cells [19, 20, 21, 22].

The numbers of multifunctional cytokine^+^ CD4 T cell that produced IFN‐γ, IL‐2 and TNF were particularly stable between days 9 and 30 post‐infection. We ruled out a role for proliferation in supporting the persistence of this population. However, it is possible that the memory T cells are a dynamic and plastic population, shifting between the cytokine^+^ and negative populations between time points. Adoptive transfer of the different cytokine^+^ and negative populations would be required to address whether cytokine producing capacity is stable and test the hypothesis that cytokine^+^ cells do indeed display enhanced survival compared to non‐cytokine^+^ T cells.

A further caveat of our model is that the detection of the cytokine producing cells involves a short *ex vivo* reactivation stage. We therefore cannot know whether the expression of these molecules is altered during the reactivation. Our previous data did, however, demonstrate that the three cytokine^+^ populations could be identified as early as 2 h after re‐activation, suggesting that these three populations are reflective of in vivo CD4 T cells with distinct phenotypes and functions [23].

We were surprised that the double and triple cytokine^+^ CD4 memory T cells did not dominate the secondary response to IAV. These cells expressed high level of transcripts for *Myc*, *Slc7a5* and the transferrin receptor, suggesting that they are poised to proliferate following TCR activation [42]. We cannot exclude that memory triple or double cytokine^+^ CD4 T cells differentiate into single IFN‐γ^+^ during the re‐infection. We think this is unlikely, however, given the stability of proportions of triple/double/single cytokine^+^ cells at days 5 and 30 following IAV re‐infection.

We identified IAV‐specific CD4 T cells using methods that restrict the population to a single immunodominant epitope or to T cells responding to numerous IAV epitopes. Our results are consistent between these methods suggesting that TCR specificity does not influence T cell survival. This conclusion is reinforced by finding the same TCR clones in several of the memory clusters within the single‐cell RNAseq dataset. We suggest that environment is more likely, therefore, to dictate cell fate, than signals through the TCR.

We have analysed IAV‐specific CD4 and CD8 T cells in the same animals highlighting some key differences. Most notably, cytokine^+^ CD8 T cells display a very limited decline in lymphoid organs reflecting similar findings in LCMV infected mice [43]. We did find consistent patterns of Ki67 expression in IAV‐specific CD4 and CD8 T cells. Single IFN‐γ^+^ CD4 and CD8 T cells were more likely to be Ki67^+^ at primary, memory and recall time points compared to triple or double cytokine^+^ T cells. These data suggest that the link between proliferation capacity and multi‐functionality is common to both CD4 and CD8 T cells. Understanding the molecular mechanisms that underlie this link could, therefore, reveal pathways that can be manipulated to enhance both the CD4 and CD8 T cell responses in the context of infectious disease and cancer or limit such responses in autoimmune pathologies.

## Materials and methods

### Animals and study design

Ten weeks old female C57BL/6 mice were purchased from Envigo. TRACE male and female and female C57BL/6 mice were maintained at the University of Glasgow under specific pathogen free conditions in accordance with UK home office regulations (Project Licenses P2F28B003 and PP1902420) and as approved by the local ethics committee. TRACE mice have been described previously [26].

### Infections

IAV was prepared and titred in MDCK cells. A period of 10–14 weeks old mice were briefly anesthetised using inhaled isoflurane and infected with 150–200 plaque forming units of Influenza A/WSN/33 (H1N1) in 20 μL of PBS intranasally (i.n.). These mice are between 18 and 25 g at the start of the experiment. Infected mice were re‐challenged with 200 PFU of Influenza A/X31 (H3N2). Infected mice were weighed daily for 14 days post‐infection. Any animals that lost more than 20% of their starting weight were humanely euthanised. TRACE mice were given Dox^+^ chow (Envigo) for a total of 12 days starting 2 days prior to infection.

### Tissue preparation

Mice were injected intravenously (i.v.) with 1 μg anti‐CD45 (30F11, either labelled with Alexa 488 or PE, Thermo Fisher) 3 min before being euthanised by cervical dislocation. Spleen and mediastinal LNs were processed by mechanical disruption. Single‐cell suspensions of lungs were prepared by digestion with 1 mg/mL collagenase D (Sigma) and 30 μg/mL DNAse (Sigma) for 40 min at 37°C in a shaking incubator followed by mechanical disruption. Red blood cells were lysed from spleen and lungs using lysis buffer (Thermo Fisher).

### Ex vivo reactivation for intracellular cytokine staining

BM DCs were prepared as described from C567BL/6 mice [23]. After 7 days, DCs were harvested, incubated overnight with IAV Ag (MOI of 0.3) prepared as described [23]. Alternatively, DCs were incubated with 10 μg/mL NP peptides (QVYSLIRPNENPAHK and ASNENMETM from JPT) for 2 h prior to co‐cultures and with indicated doses shown in Fig. 1E and F. Single‐cell suspensions of *ex vivo* organs were co‐cultured with DCs in complete RMPI at a ratio of approximately 10 T cells to 1 DC in the presence of Golgi Plug (BD Bioscience). Co‐cultures were incubated at 37°C, 5% CO_2_ for 6 h.

### Flow cytometry staining

Single‐cell suspensions were stained with PE or APC‐labelled IA^b^/NP_311–325_ or APC labelled D^b^/NP_368–374_ tetramers (NIH tetramer core) at 37°C, 5% CO_2_ for 2 h in complete RPMI (RPMI with 10% foetal calf serum, 100 μg/mL penicillin–streptomycin and 2 mM l‐glutamine) containing Fc block (24G2). Anti‐CX3CR1 BV711 (SA011F11, BioLegend) was added with the MHC tetramers. Surface antibodies were added and the cells incubated for a further 20 min at 4°C. Antibodies used were anti‐CD4 APC‐Alexa 647 (RM4‐5, Thermo Fisher), anti‐CD8 BUV805 (53‐6.7, BD Bioscience), anti‐CD44 BUV395 (IM7, BD Bioscience), anti‐PD‐1 PeCy7 (29F.1A12, BioLegend), anti‐PD1 BV605 (29F.1A12, BioLegend), anti‐ICOS PerCP‐Cy5.5 (7E.17G9, BioLegend), anti‐CD127 APC (A7R34, Thermo Fisher), CD103 PeCy7 (2E7, BioLegend), anti‐CD69 PerCP‐Cy5.5 (H1.2F3, Thermo Fisher) and ‘dump’ antibodies: B220 (RA3‐6B2), F4/80 (BM8) and MHCII (M5114) all on eFluor‐450 (Thermo Fisher). Cells were stained with a fixable viability dye eFluor 506 (Thermo Fisher). For normalisation of CD127 and Bcl2 expression, the MFI of the EYFP^+^ cells was divided by the MFI of naïve (CD44lo) CD4 T cells in the same animal in the same organ.

For intracellular staining, cells were fixed with cytofix/cytoperm (BD Bioscience) for 20 min at 4°C and stained in permwash buffer with anti‐cytokine antibodies for 1 h at room temperature anti‐IFN‐γ PE (XMG1.2, Thermo Fisher) or Brilliant violet 785 (XMG1.2, BioLegend), anti‐TNF Alexa‐Fluor‐488 (MP6‐XT22, Thermo Fisher) or Brilliant Violet 605 (MP6‐XT22, BioLegend), anti‐IL‐2 APC (JES6‐5H4, Thermo Fisher) or Brilliant violet 711 (JES6‐5H4, BioLegend), Bcl2 PeCy7 (Blc/10C4, BioLegend), anti‐Ki67 PeCy7 (16A8, BioLegend) and cells washed with permwash buffer. To detect Myc, cells were first stained with unlabelled anti‐Myc (D84C12, Cell Signalling Technologies), cells washed with permwash and then stained with PE‐anti‐rabbit (Cell signalling Technologies). Stained cells were acquired on a BD LSR or Fortessa and analysed using FlowJo.

### BD Rhapsody single‐cell RNA‐seq

CD4 T cells from spleens and lungs of IAV infected TRACE mice were isolated by CD4 negative selection following the manufacturer's instructions (StemCell, CD4 Isolation Kit). The cells were activated with IAV‐Ag‐DCs for 4 h and then stained with anti‐CD4 APC‐Alexa 647, anti‐CD44‐PerCP‐Cy5.5 (IM7, Thermo Fisher), anti‐MHCII‐e450, anti‐B220‐e450, CD8‐e450 (53‐6.7, Thermo Fisher), F4/80‐e450 and CD45 Sample Tags to enable multiplexing (BD Bioscience,) and AbSeq antibodies to ICOS (AMM2072) and PD1 (AMM2138). CD44^hi^/EYFP^+^ and CD44^lo^/EYFP‐negative cells from combined spleen or lung samples were FACS sorted on an ARIA IIU and cells transferred into BD Rhapsody Sample Buffer and loaded onto a scRNA‐seq Rhapsody Cartridge (5000 EYFP‐negative CD44^lo^ spleen cells; 5000 EYFP + CD44^hi^ spleen cells; and 1000 EYFP + CD44^hi^ lung cells. Manufacturer's instructions (mouse VDJ CDR3 library preparation) were followed to prepare libraries for sequencing with the BD Rhapsody Immune response mouse targeted panel, additional custom made primers (Excel file 1 in the Supporting Information), sample Tags and VDJ CDR3. Pair‐end sequencing was performed by Novogene on an Illumina MiSeq PE250.

### Single‐cell RNA‐seq analysis

Data were initially processed on Seven Bridges and then analysed predominantly using Seurat (V4.2.0) [44] and scRepertoire (V1.7.2) [45]. Briefly, to perform dimensionality reduction, count data were normalised using scTransform prior to principal component analysis, Uniform Manifold Approximation and Projection dimensional reduction, Nearest‐Neighbour graph construction and cluster determination within the Seurat package and dimensionality reduction performed. Log normalising and scaling of the count data were conducted prior to differential gene expression analysis. To test for differential gene expression, the FindAllMarkers function was used (min.pct = 0.4 and minLogFC = 0.25, model.use = MAST). Only genes with a Bonferroni corrected *p* value <0.05 were considered statistically different. For TCR analysis, clonal expansion was calculated based on the same TCR being detected once (Single Clone) or multiple times within the same animal. All TCR analysis utilised stock or customised code from the scRepertoire package. Further packages used for data analysis, organisation and visualisation included workflowr [46], dplyr, ggplot2, cowplot, ggvenn, circlize [47], plot1cell [48] and ComplexHeatmap [49]. All code used can be found at https://github.com/JonathanNoonan/Westerhof.

### Statistical analysis

All data other than scRNAseq were analysed using Prism version 9 software (GraphPad). Differences between groups were analysed by unpaired ANOVAs, *t*‐tests or Mann–Whitney test as indicated in figure legends. In all figures, ‘*’ represents a *p* value of <0.05; **: *p* < 0.01, ***: *p* < 0.001, ****: *p* < 0.0001.

## Conflict of interest

The authors have no conflict of interest to declare.

## Author contributions

Conception; investigation; formal analysis; visualization; writing – original draft and reviewing/editing: Lotus M. Westerhof. Formal analysis; data curation; software; visualization; writing – reviewing and editing: Jonathan Noonan. Investigation; formal analysis; writing – reviewing and editing: Kerrie E. Hargrave, Elizabeth T. Chimbayo and Zhiling Cheng. Investigation; writing – reviewing and editing: Thomas Purnell. Formal analysis; writing – reviewing and editing: Mark R. Jackson. Software; writing – reviewing and editing: Nicholas Borcherding. Supervision; project management; funding acquisition; conception; investigation; formal analysis; visualization; writing – original draft and reviewing: Megan K. L. MacLeod.

### Peer review

The peer review history for this article is available at https://publons.com/publon/10.1002/eji.202350559.

**Abbreviations**
CDR: Complementarity‐determining region
DCs: dendritic cells
dLN: draining LN
IAV Ag: IAV antigens
IAV: influenza A virus
IFN: Interferon
IL: Interleukin
NP: nucleoprotein
UMAP: Uniform Manifold Approximation and Projection

## Supporting information

Supporting Information

Supporting Information

Supporting Information

## Data Availability

The scRNA‐seq data have been deposited at GEO and are publicly available as of the date of publication; GSE220588. All code used can be found at https://github.com/JonathanNoonan/Westerhof_2023.
