# Supplementary material for: Multifunctional cytokine production marks influenza A virus‐specific CD4 T cells with high expression of survival molecules
Source: Eur J Immunol. 2023 Jul 30;53(11):2350559. doi: 10.1002/eji.202350559 (PMC10947402; doi:10.1002/eji.202350559)
Supplement: Supplementary file 1 — Supporting Information [file EJI-53-0-s002.pdf]

Supplementary Figure 1: Gating strategies for identification of IAV specific CD4 and CD8 T cells and example staining for surface markers on lung T cells

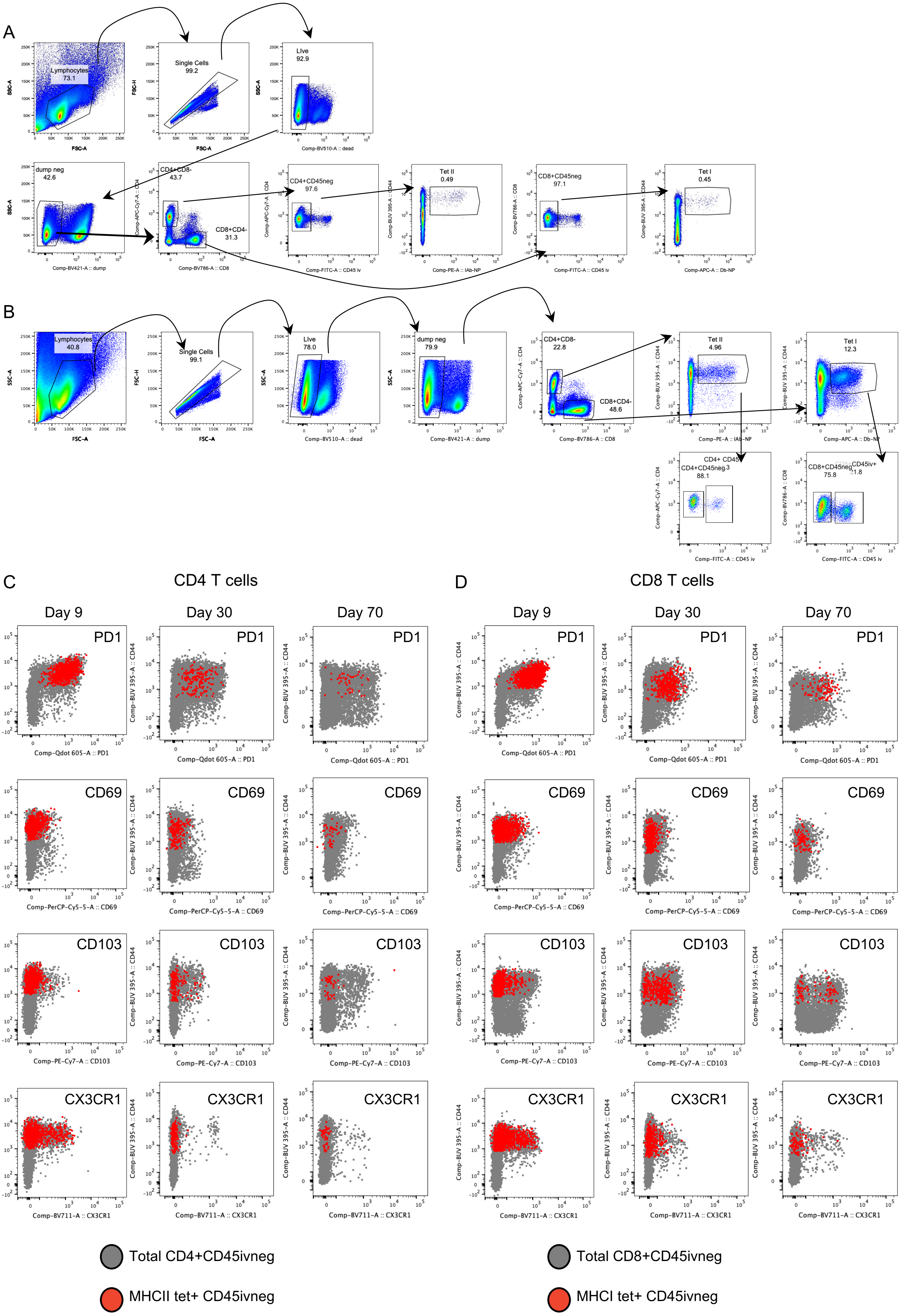

Supplementary Figure 1
Example gating of IAV specific CD4 and CD8 T cells identified by MHC tetramers

C57BL/6 mice were infected i.n. with IAV on day 0 and injected i.v. with fluorescently labelled anti-CD45 3 minutes prior to removal of organs for analysis. Single cell suspensions of spleen (A) and lung (B) are shown at day 9 of infection. A, shows example gating used in most figures in which we gate on CD45 i.v. negative tetramer+ or cytokine+ T cells. Example FACS plots of splenic CD45iv negative MHC tetramer+ cells CD4 (C) and CD8 (D) (shown in red) in comparison to total CD45iv negative T cells (grey).

Supplementary Figure 2: IL-2 and TNF+ CD4 and CD8 T cells show minimal decline between day 9 and day 30 in secondary lymphoid organs

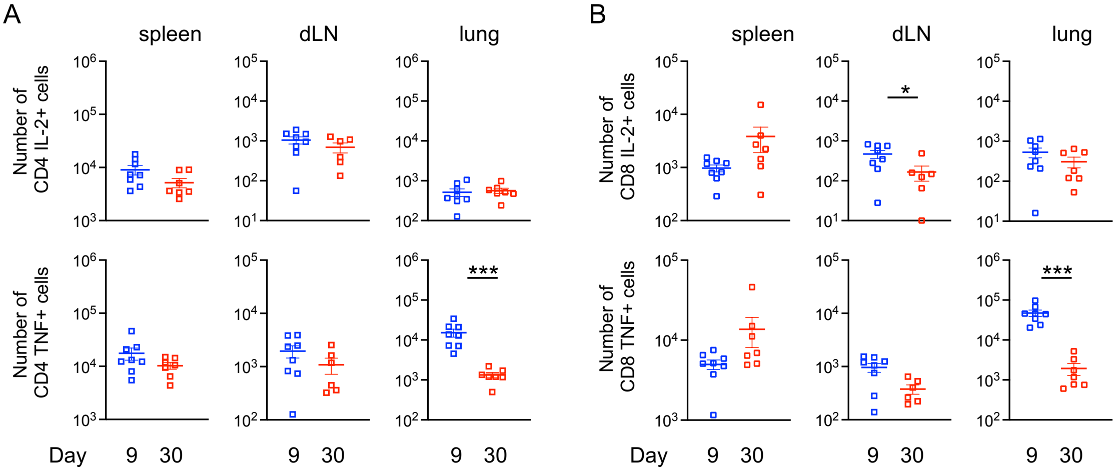

Supplementary Figure 2

IL-2 and TNF+ CD4 and CD8 T cells show minimal decline between day 9 and day 30 in secondary lymphoid organs

C57BL/6 mice were infected i.n. with IAV on day 0 and injected i.v. with fluorescently labelled anti-CD45 (CD45iv) 3 minutes prior to removal of organs. Single cell suspensions of spleens, mediastinal draining lymph node (dLN), and lung were examined after 9 or 30 days and activated with IAV-peptide loaded DCs. Numbers of IL-2 or TNF+ CD4 (A) or CD8 T cells (B) were calculated. Data are from two independent time course experiments with a total of 7-8 mice/time point. Y-axis set at the limit of detection and errors are SEM. Significance tested by a Mann-Whitney, *: $p<0.05$, ***:$p<0.001$.

Supplementary Figure 3: TRACE mice enable identification of CD4 T cells responding to IAV infection

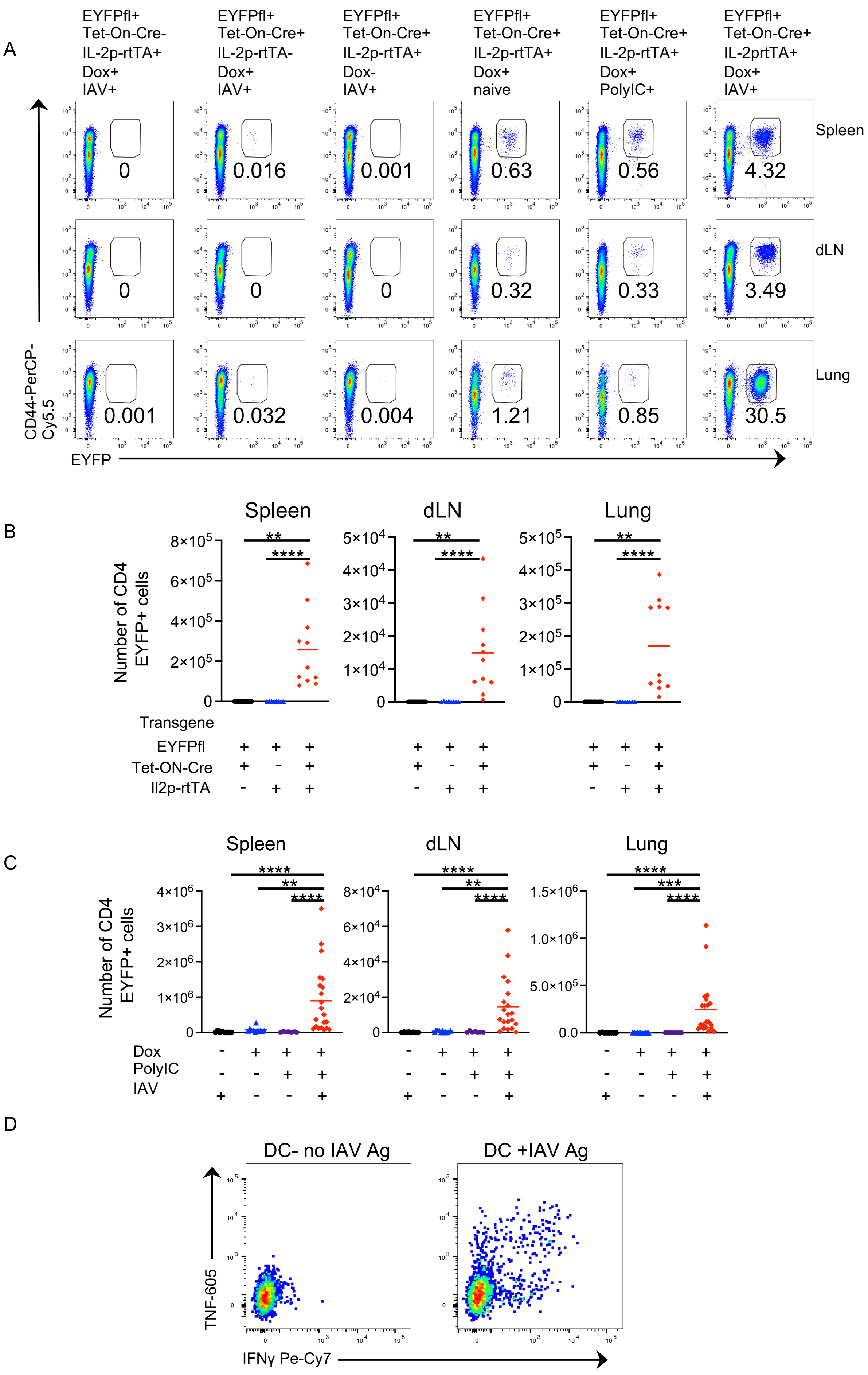

Supplementary Figure 3

TRACE mice enable identification of CD4 T cells responding to IAV infection

TRACE mice that are positive for all three transgenes and double transgenic mice expressing STOP floxed EYFP (EYFPfl) and either tet-ON-Cre or the IL-2 promoter driving rtTA (IL-2p-rtTA) were given doxycycline (Dox) diet for a total of twelve days starting two days prior to intranasal infection with IAV or instillation of 20μg of PolyIC intranasally as indicated. In A, cells are gated as in Supplementary Figure 1A on CD4+ live lymphocytes that are negative for CD8, B220, MHCII and F4/80 in the indicated organ 8 days after infection/polyIC treatment and the numbers show the percentages of EYFP+ cells within the gate. In B, mice with the indicated transgenes were given dox diet and the numbers of EYFP+ CD4 T cells examined 8-12 days following treatment, data are combined from 3 independent experiments with 2-5 mice per experiment. In C, TRACE mice were treated/infected as indicated and the numbers of EYFP+ CD4 T cells examined 8-12 days following infection, data are combined from 4 independent experiments with 2-10 mice per experiment. Significance tested by a Kruskal-Wallis test followed by a Dunn's multiple comparison test: *: $p<0.05$, **:$p<0.01$, ***:$p<0.001$, ****:$p<0.0001$. D shows example staining from a day 8 IAV infected mouse, the cells on the left were co-cultured with DCs that had not received IAV-Ag, the plot on the right shows cells from the same mouse co-cultured with IAV-Ag+ DCs for 6 hours. Cells are gated on live single CD4+ EYFP+ cells that are CD45iv negative.

Supplementary Figure 4: TRACE mice enable identification of cytokine+ and negative T cells

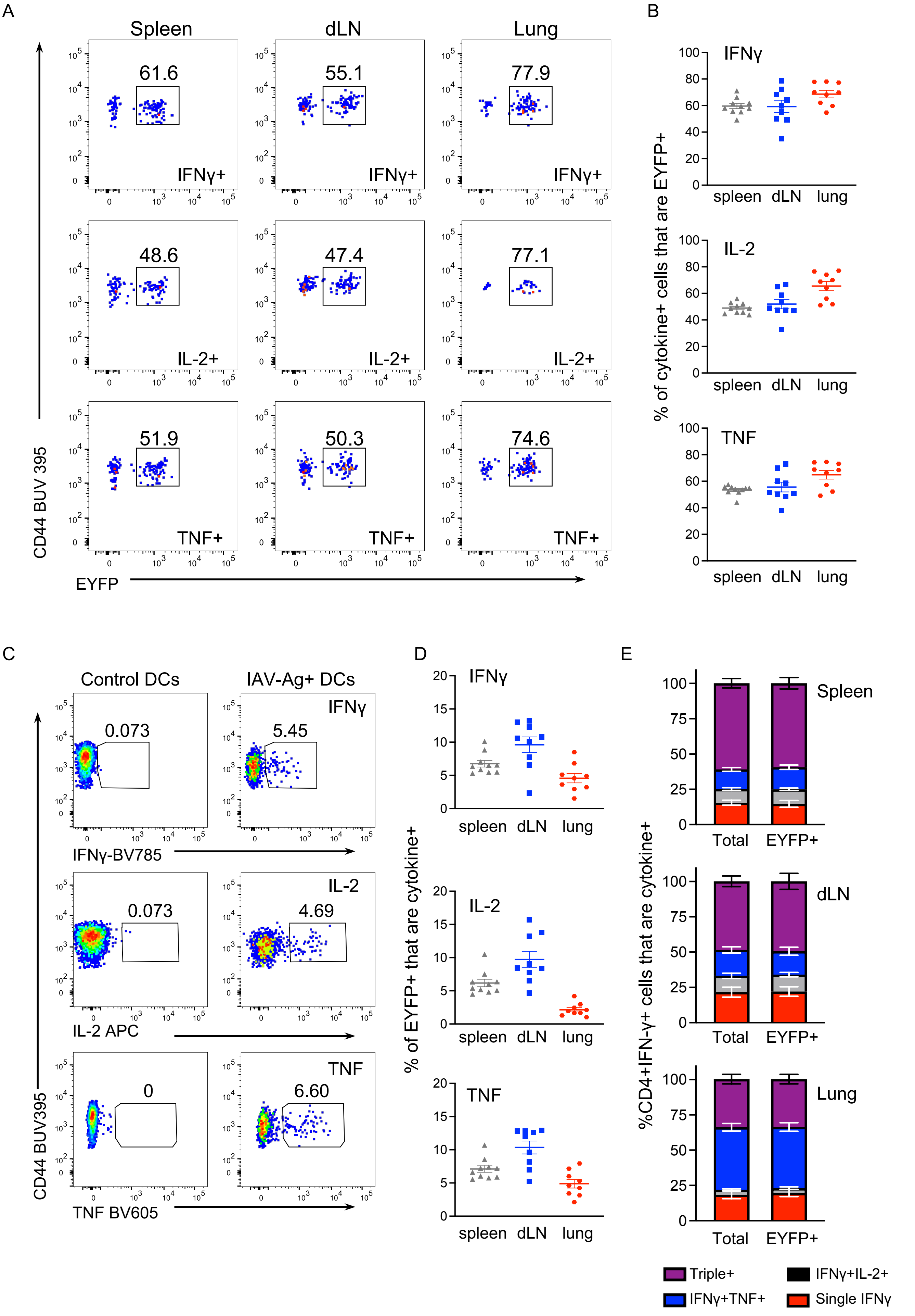

Supplementary Figure 4:

TRACE mice enable identification of cytokine+ and negative CD4 T cells

TRACE mice were infected i.n. with IAV on day 0 and injected i.v. with fluorescently labelled anti-CD45 3 minutes prior to removal of organs at day 14 post-infection. Single cell suspensions of spleens, mediastinal draining lymph node (dLN), and lung were activated by DCs incubated with IAV-Ag preparation or control DC. The percentages of CD45iv negative IFNγ, IL-2 and TNF+ cells that were EYFP were determined (A-B). Alternatively, the percentages of EYFP+ cells producing IFNγ, IL-2 or TNF in response to control DC (no IAV Ag) or IAV-Ag+ DCs were analysed (C-D) and the percentages of total IFNγ+ or EYFP+ IFNγ+ CD4 T cells that also produced IL-2 and/or TNF were determined (E). Some lymph nodes and lungs are excluded as the number of cytokine+ cells within the FACS plot was less than 10. A shows representative FACS plots gated on live CD45iv negative, CD4+cyokine+ and the numbers are the percentages of cells within the EYFP+ gate. C shows spleen cells gated on live CD454iv negative CD4+ EYFP+ cells and the numbers are the percentages of these cells that are cytokine+. In B and D, data are combined from two experiments with 4-6 mice, each point is a mouse and the horizontal line is the mean, errors are SEM. In E, data are from the same experiments and error bars are SEM.

Supplementary Figure 5: Detection of IFNγ Ki67+ cells by flow cytometry

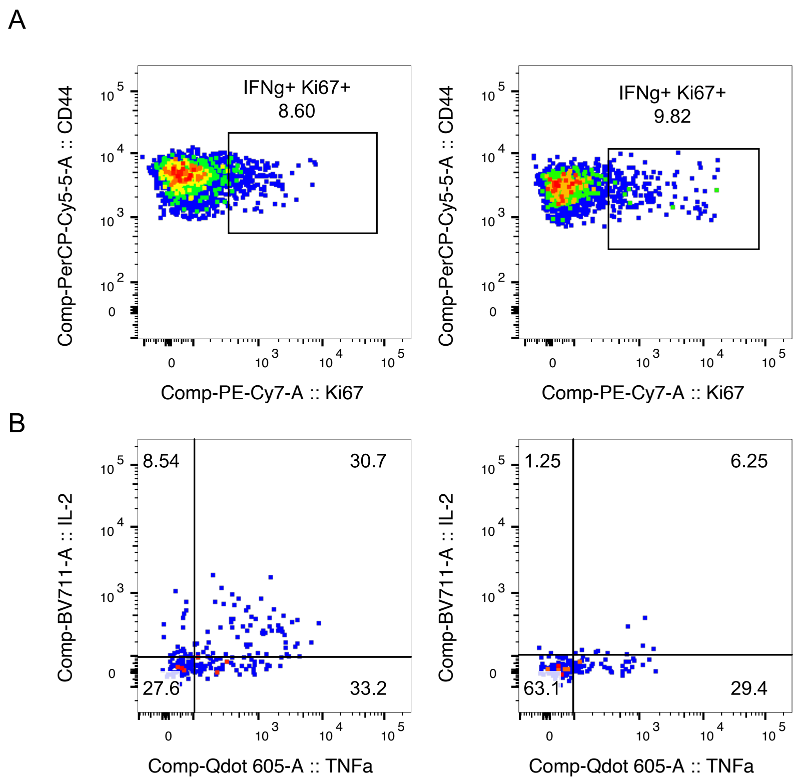

Supplementary Figure 5

Detection of IFNγ Ki67+ cells by flow cytometry

C57BL/6 mice were infected i.n. with IAV on day 0 and injected i.v. with fluorescently labelled anti-CD45 (CD45iv) 3 minutes prior to removal of organs. At day 9 post-infection, single cell suspensions of spleens were co-cultured with IAV-Ag+ DCs for 6 hours. In A, cells are gated as in Supplementary Figure 1A and on CD4 or CD8 T cells that are IFNγ+. In the bottom panel, cells are also gated on the Ki67+ population shown in A. The numbers show the percentage within the indicated gates or quadrants. Data are representative of the experiments shown in Figure 3.

Supplementary Figure 6: Example of sorted memory and naïve CD4 T Cells

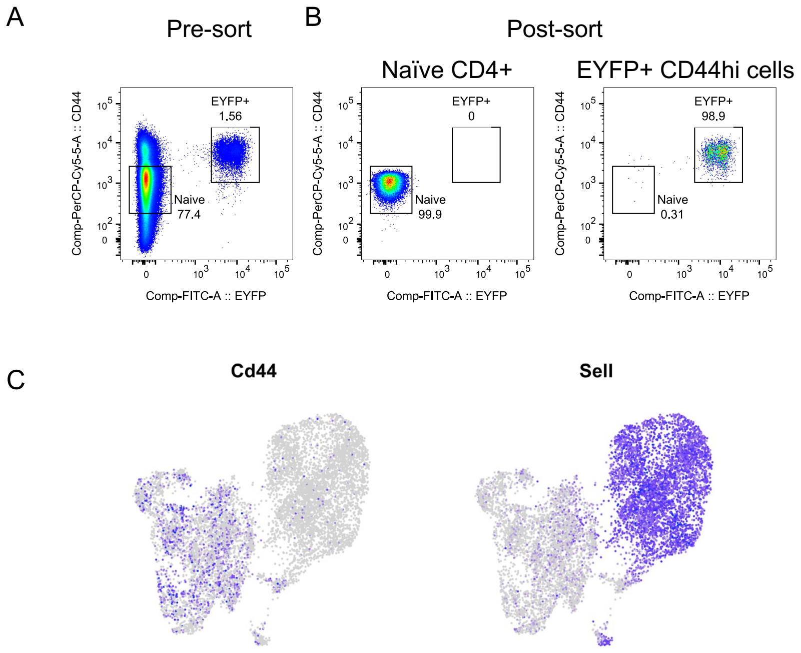

Supplementary Figure 6

Example data from sorted EYFP negative naïve and EYFP+ memory CD4 T cells

TRACE mice were infected with IAV and injected with anti-CD45 3 minutes prior to removal of spleens and lungs. Isolated CD4 T cells were activated for 4 hours by co-culture with IAV-Ag DCs and the CD45iv negative CD4+CD44^{hi}EYFP+ cells (spleens and lung) and CD4+CD44^{lo}EYFPnegative (spleens) cells were FACS sorted. In A, FACS plots are gated on live CD4+ single lymphocytes negative for CD45iv, B220 and MHCII and show cells prior to the sort with sort gates shown. In B, cells are gated on the indicated cells post-sort. A-B are from a practice sort prior to isolation of the cells for scRNAseq transcriptomic analysis. In C, the expression of *Cd44* and *Sell* (which codes for CD62L) are shown overlaid on the UMAP described in Figure 4A.

Supplementary Figure 7: scRNAseq reveals heterogeneity in the memory CD4 T cell pool

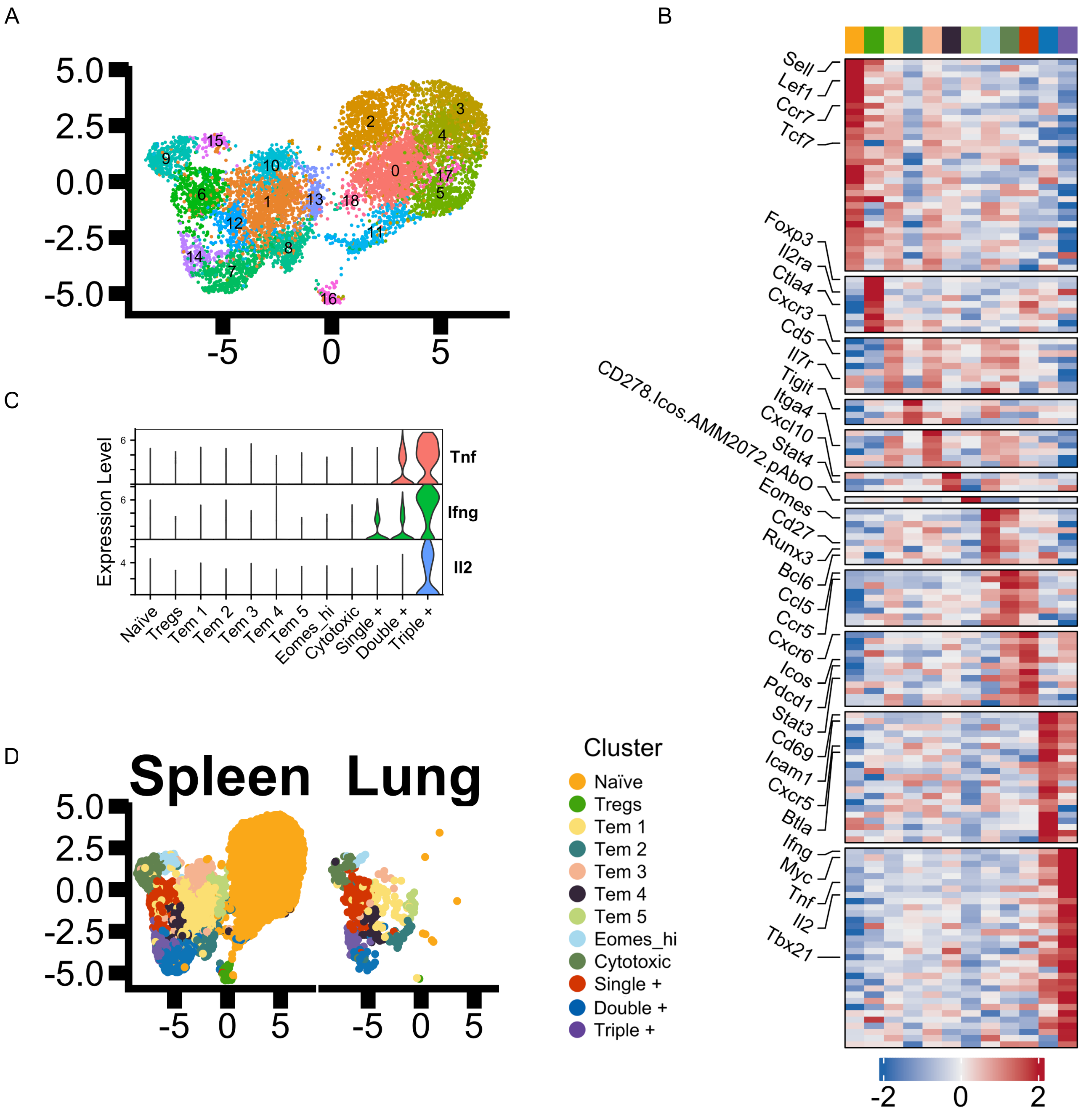

Supplementary Figure 7

scRNAseq reveals heterogeneity in the memory CD4 T cell pool

TRACE mice were infected with IAV and injected with anti-CD45 3 minutes prior to removal of spleens and lungs. Isolated CD4 T cells were activated for 4 hours by co-culture with IAV-Ag DCs and the CD45iv negative CD4+CD44^{hi}EYFP+ cells (spleens and lung) and CD4+CD44^{lo}EYFPnegative (spleens) cells were FACS sorted and their transcriptomes examined by scRNAseq. UMAP of eight naïve clusters, one Treg cluster, and 10 memory clusters (A). DEGs in main clusters identified by comparison between the indicated populations and all other clusters (B). Where genes were differentially expressed in multiple clusters, the gene was visualised in the cluster with the highest fold change; for a list of all DEGs see Supplementary Table 1. Expression of *Tnf*, *Ifng* and *Il2* by each cluster (C). UMAP of analysed cells displaying cells from the spleen and lung separately (D).

Supplementary Figure 8: Triple cytokine+ T cells make more cytokine on a per cell basis than single cytokine+ T cells

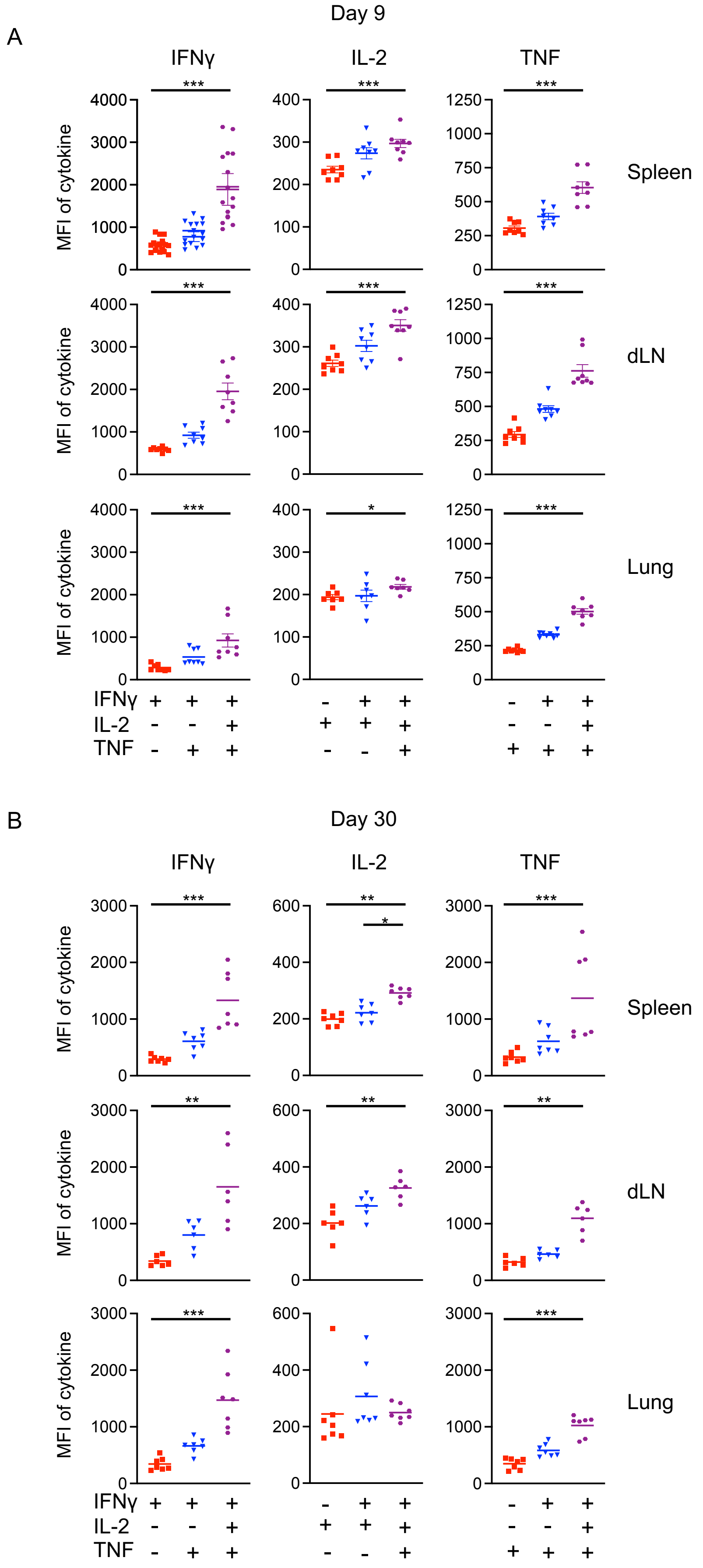

Supplementary Figure 8

Triple cytokine+ CD4 T cells produce more cytokine on a per cell basis than single cytokine+ T cells

C57BL/6 mice were infected i.n. with IAV on day 9 or day 30 and injected i.v. with fluorescently labelled anti-CD45 3 minutes prior to removal of organs for analysis. Single cell suspensions of spleens, mediastinal draining lymph node (dLN), and lung were activated by DCs incubated with IAV-Ag. CD45iv negative IAV specific CD4 T cells cytokine+ at day 9 (A) or day 30 (B) T cells were detected by flow cytometry. Each symbol represents a mouse and the line shows the mean of the group with SEM shown. Significant differences were assessed by Friedman's multiple comparison test followed by multiple comparisons with Dunn's multiple comparison test; *: $p<0.05$, **: $p<0.01$, ***: $p<0.001$.

Supplementary Figure 9: Single IFNγ+ CD4 memory T cells express higher levels of PD1 and ICOS than triple cytokine+ cells

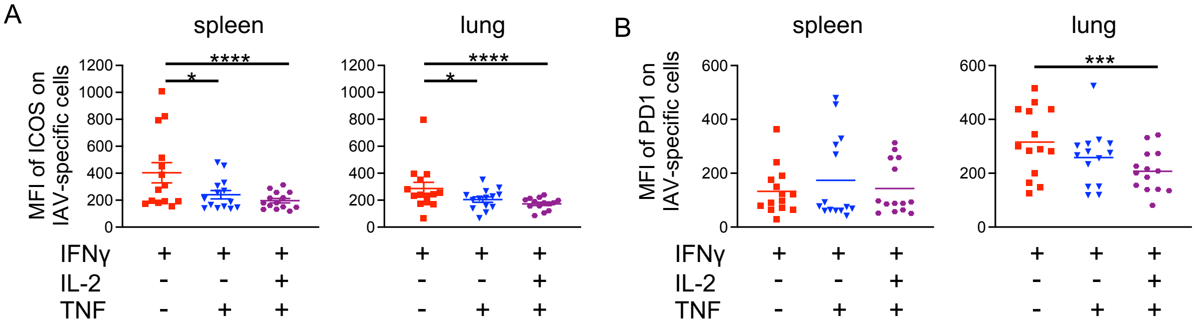

Supplementary Figure 9

Single IFNγ+ CD4 memory T cells express higher levels of PD1 and ICOS than triple cytokine+ cells

C57BL/6 mice were infected i.n. with IAV on day 0 and injected i.v. with fluorescently labelled anti-CD45 3 minutes prior to removal of organs at day 40. Single cell suspensions of spleens and lung were activated by DCs incubated with IAV-Ag. CD45iv negative cytokine+ CD4 T cells were detected by flow cytometry to detect ICOS (A) and PD1 (B) expression. Data are from two separate experiments. Each symbol represents a mouse and the horizontal line shows the mean of the group. Significance tested via paired Friedman analysis with Dunn's multiple comparison test *: $p<0.05$, ***:$p<0.001$, ****:$p<0.0001$.

Supplementary Figure 10: Previous infection with IAV leads to a protective response following re-challenge infection but no changes in the proportion of cytokine+ populations

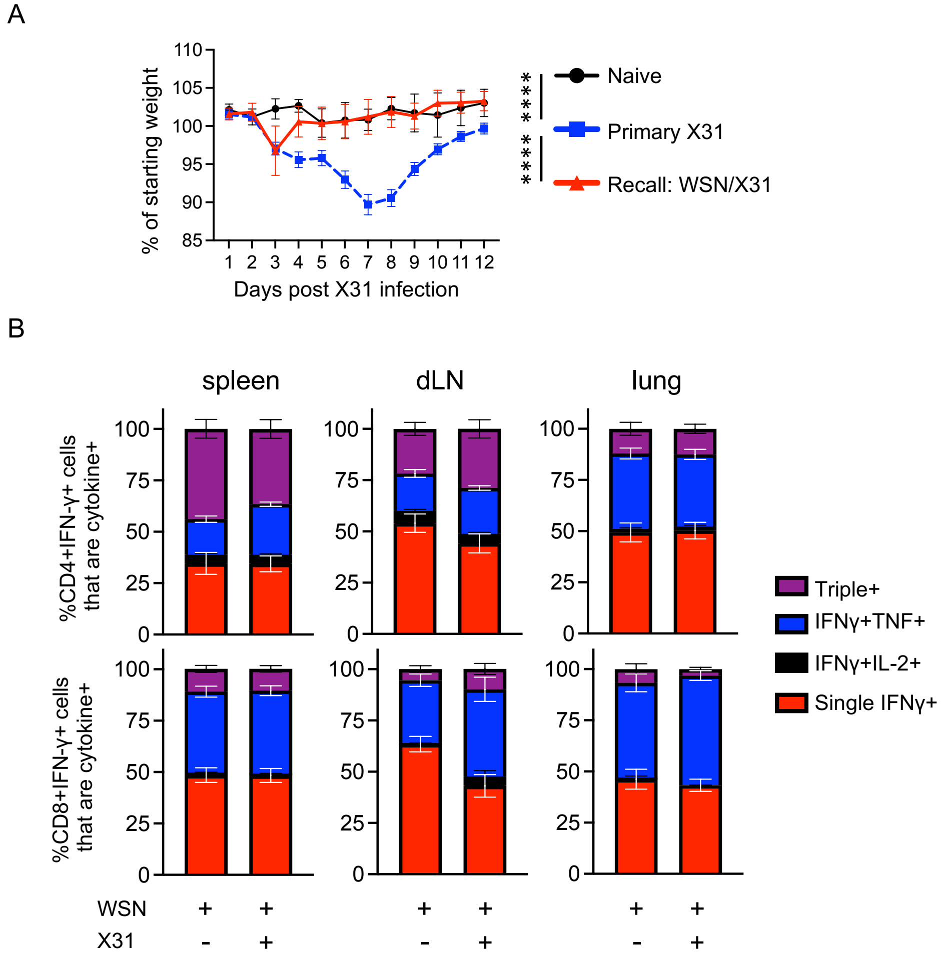

Supplementary Figure 10

Previous infection with IAV leads to a protective response following re-challenge infection but no changes in the proportion of cytokine+ populations

C57BL/6 mice were infected i.n. with WSN IAV on day -30 and then infected with X31 on day 0. Controls were age-matched naïve animals or naïve mice infected with X31 IAV on day 0. Mice were weighed and the difference in weight loss calculated by measuring the area and the curve (A). On day 5 after the challenge infection, a separate cohort of mice were injected i.v. with fluorescently labelled anti-CD45 3 minutes prior to removal of organs for analysis. Single cell suspensions of spleens, mediastinal draining lymph node (dLN), and lung were activated by bmDCs incubated with IAV-Ag. The proportions of IFNγ+ CD4 and CD8 T cells expressing IL-2 and TNF were calculated (B). In A, naïve animals are from one experiment (4 naïve animals) and infected animals combined from two experiments with a total of 12 primary infected animals and 9 re-infected animals. The areas under the curves were compared by ANOVA followed by a Tukey's multiple comparison test with ****:$p<0.0001$. In B, data are from two experiments with a total of 7-8 mice/group, error bars are SEM.
